# A rare case report on esophageal squamous cell carcinoma metastatic to the pancreas

**DOI:** 10.3389/fonc.2025.1602987

**Published:** 2025-07-24

**Authors:** Inderpreet Bhatti, Robert E. Rogers, Benjamin L. Witt, Chris Nevala-Plagemann, Jeffrey Patterson-Fortin

**Affiliations:** ^1^ Division of Oncology, Huntsman Cancer Institute, University of Utah, Salt Lake City, UT, United States; ^2^ Department of Pathology, University of Utah, Salt Lake City, UT, United States

**Keywords:** esophageal cancer, squamous cell carcinoma, pancreatic metastasis, tumor origin identification, rare metastasis sites, tumor dissemination

## Abstract

Pancreatic metastasis of esophageal squamous cell carcinoma is extremely rare. We describe a case of man aged 66 years who presented with a 3-month history of dysphagia. Esophagogastroduodenoscopy demonstrated an esophageal mass. Additional asymptomatic solitary lung and pancreatic masses were observed in the staging work-up for esophageal cancer, concerning for metastatic versus synchronous primary malignancy. Computed Tomography guided needle biopsy of the lung and pancreatic masses was performed. Given the difficulty to definitively histologically confirm both the lung and the pancreatic masses were metastasis from the primary esophageal cancer, next generation sequencing and tumor origin testing was performed. Tumor origin testing was indeterminate with the molecular profile inconclusive. Treatment was initiated with chemotherapy and immunotherapy.

## Background

Esophageal cancer is a common cancer, with the majority being either squamous cell or adenocarcinomas, and a common cause of death worldwide (1). The incidence of esophageal cancer varies globally by region with the incidence declining in North America with 6643 cases of esophageal squamous cell carcinoma in 2020 (1). Esophageal cancer typically metastasizes to the liver, lungs, and bone, whereas pancreatic metastases are extremely rare. Indeed, review of the literature revealed that metastatic lesions of the pancreas are rare and account for approximately 2-5% of pancreatic tumors (2). In addition, review of the literature revealed that less than 5% of pancreatic metastases are from primary esophageal cancers (3). Our case represents an extremely rare presentation of esophageal squamous cell carcinoma with pancreatic metastasis.

## Case presentation

**Figure 1:**
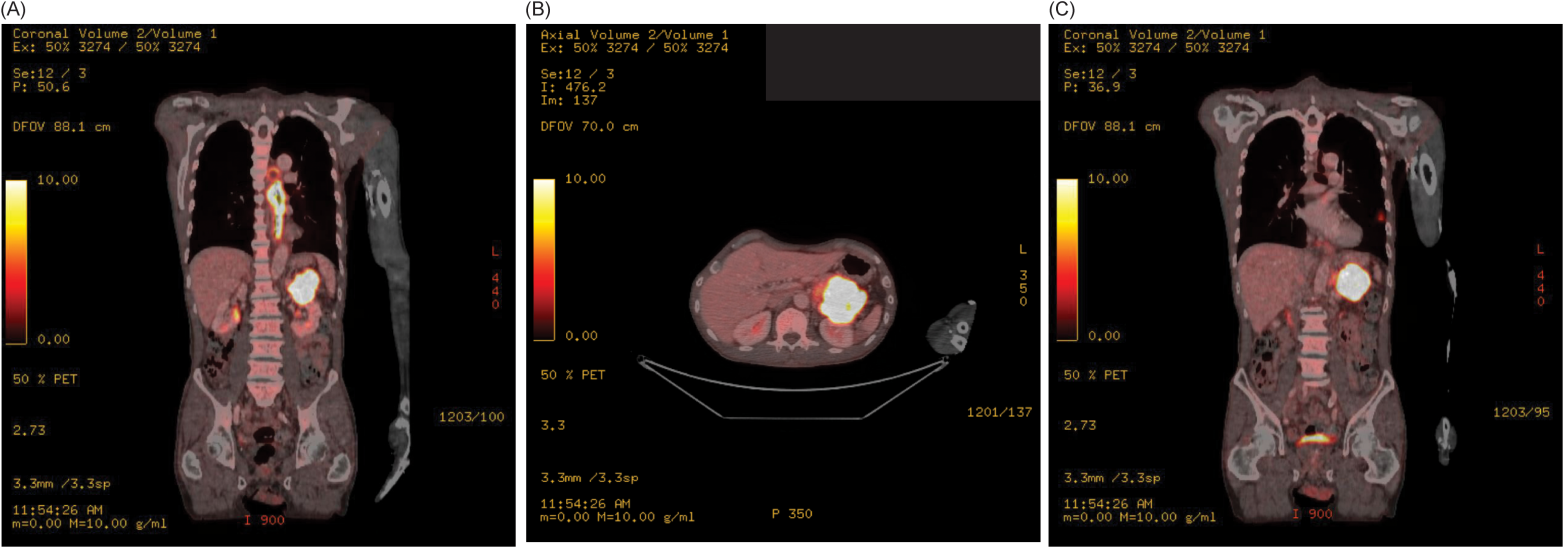
**(A)** Staging Positron Emission Tomography/Computed Tomography scan demonstrates a diffusely thickened esophagus with FDG avidity in the upper esophagus at the level of the aortic arch to the gastroesophageal junction, and FDG avid pancreatic tail mass, in the coronal plain. **(B)** Staging Positron Emission Tomography/Computed Tomography demonstrates FDG avid pancreatic tail mass in the axial plane. **(C)** Staging Positron Emission Tomography/Computed Tomography scan demonstrates a left lower lobe superior segment FDG avid pulmonary nodule in the coronal plain.

A 66-year-old man with minimal past medical history presented to his primary care physician for a 3-month history of progressive dysphagia, now intolerant of solid foods, and accompanying 15 lbs. weight loss. Social history was notable for heavy alcohol use (14 standard drinks per week) and being a never smoker. No family history of cancer. Physical exam was notable for cachexia but no abdominal tenderness, rebound, or palpable masses. To evaluate the patient’s dysphagia and weight loss, an esophagogastroduodenoscopy (EGD) was performed which demonstrated an esophageal stricturing mass which did not allow passage of the scope at approximately 25–30 cm from the incisors. The mass was biopsied, and pathology was consistent with poorly differentiated invasive squamous cell carcinoma. Staging Positron Emission Tomography/Computed Tomography (**Figure 1**) demonstrated a diffusely thickened esophagus with fluorodeoxyglucose (FDG) avidity in the upper esophagus at the level of the aortic arch with a standardized uptake value (SUV) of 7.9, with the remainder of the esophagus below the aortic arch thickened and FDG avid to the gastroesophageal junction with a SUV of 16.2, a left lower lobe superior segment fluorodeoxyglucose (FDG) avid pulmonary nodule measuring 1.2 cm with a SUV of 5.3 concerning for metastatic disease, and a pancreatic tail FDG avid mass measuring 7.5 cm with SUV of 32 concerning for metastatic disease versus synchronous primary malignancies.

### Differential diagnosis

The differential diagnosis included the following:

- Metastatic esophageal squamous cell carcinoma,
- Metastatic lung squamous cell carcinoma,
- Metastatic pancreatic squamous cell carcinoma,
- Synchronous primary malignancies.

### Investigations

**Figure 2:**
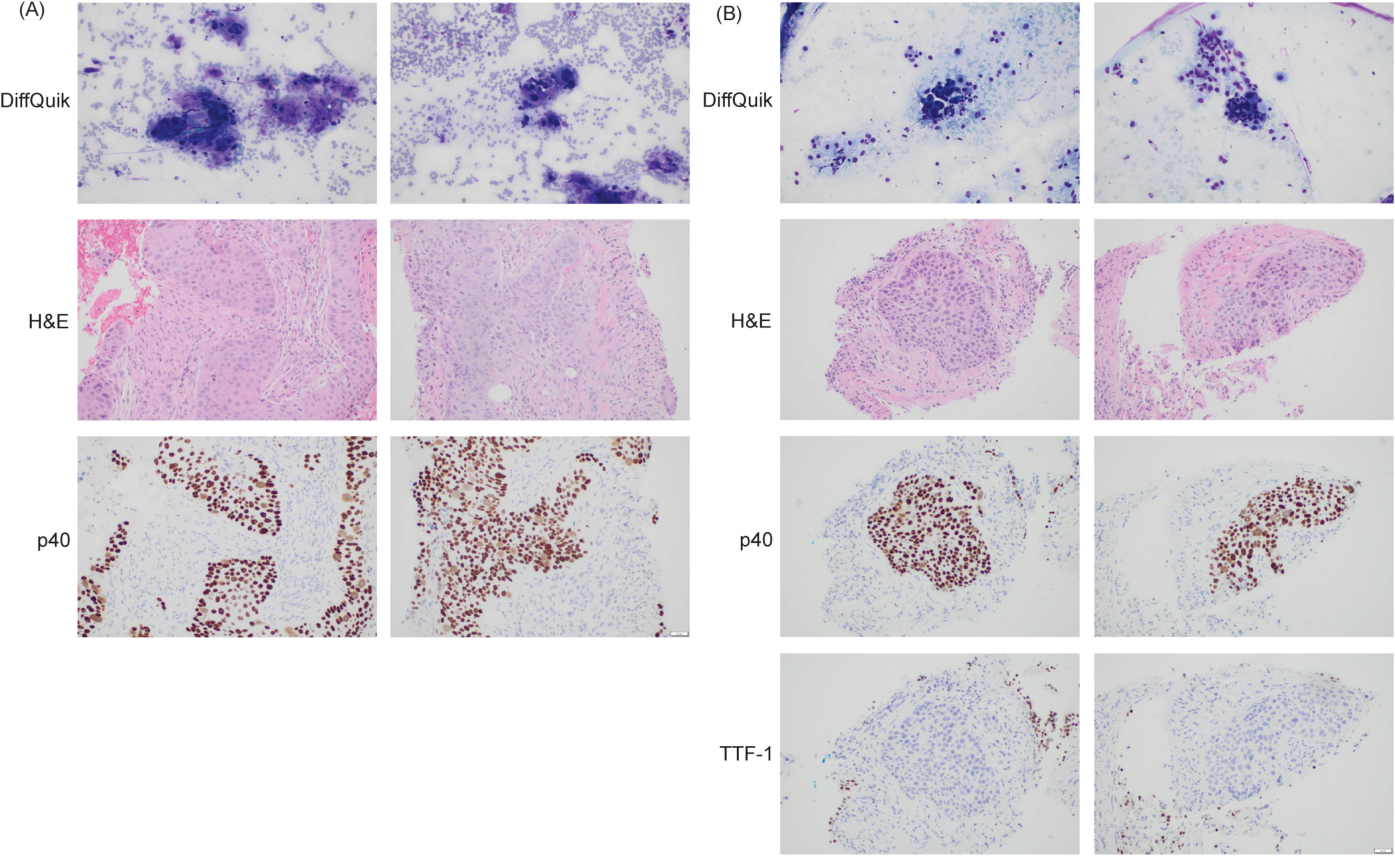
**(A)** Histological examination of pancreas biopsy. p40 is a marker of squamous cell carcinoma (positive). Magnification: 200x, acquired with Olympus DP23 camera on Olympus BX43 microscope using cellSens Entry Software. **(B)** Histological examination of lung biopsy. p40 is a marker of squamous cell carcinoma (positive). TTF-1 is a marker for primary lung adenocarcinoma (negative). Magnification: 200x, acquired with Olympus DP23 camera on Olympus BX43 microscope using cellSens Entry Software.

To resolve the question of metastatic disease versus synchronous primary malignancies, interventional radiology guided biopsy of the lung and pancreatic masses was performed and compared to the biopsy from the EGD. Histology (**Figure 2**) of the lung and pancreatic biopsies were similar in appearance. The cytologic preparations demonstrated clusters of epithelioid cells with anisonucleosis, irregular contours, and moderate nuclear-to-cytoplasmic ratios with background parenchyma and necrotic debris. Immunohistochemical staining demonstrated that the tumor cells were positive for p40, negative for TTF-1 and with a PD-L1 combined positive score of 11-20. Taken together the findings are those of squamous cell carcinoma at both sites (pancreatic tail and left lung). Subsequently, these biopsies were compared to the esophageal mass biopsy and the malignant cells were histologically similar, suggesting metastasis from a primary esophageal squamous cell carcinoma (**Figure 2**). Given the difficulty to definitively histologically confirm both the lung and the pancreatic masses were metastasis from the primary esophageal cancer, next generation sequencing and tumor origin testing (Tempus) was performed. The Tempus Tumor Origin can determine the histological subtype of 68 different malignancies with 91% accuracy using RNA expression data (4). Unfortunately, Tumor Origin testing revealed an indeterminate result with the molecular profile inconclusive favoring either gastroesophageal squamous cell carcinoma, lung squamous cell carcinoma, or pancreatic adenocarcinoma.

### Treatment and follow-up

**Figure 3:**
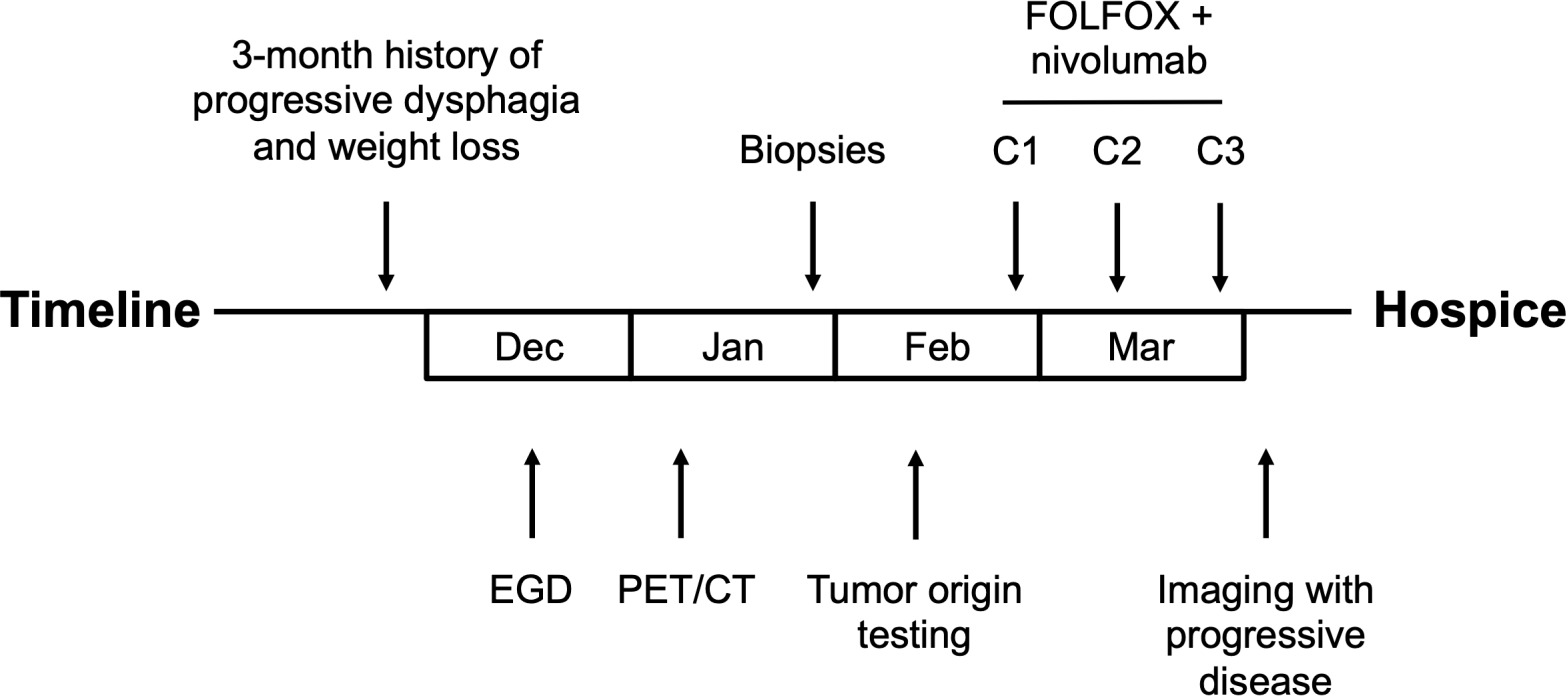
Timeline of case report.

First-line treatment was initiated with nivolumab in combination with 5-fluorouracil and oxaliplatin based on the reported survival benefit over chemotherapy alone from the CheckMate 648 trial (5). The patient received 3 cycles of FOLFOX plus nivolumab. FOLFOX plus nivolumab was dosed as follows: nivolumab 240 mg intravenous (IV); oxaliplatin 85 mg/m2 IV; and 5-fluorouracil 1,200 mg/m2/day over 48 hours IV, every two weeks. Prior to the planned fourth cycle of treatment, the patient was admitted with hypernatremia. Imaging during this admission demonstrated progressive disease with worsening bilateral pulmonary metastatic disease, increasing mediastinal and paraesophageal lymphadenopathy and pericardial disease, and increased hepatic and right perinephric metastases. Decision was made to transition to hospice (**Figure 3**).

## Discussion

We describe the case of a patient initially diagnosed with esophageal squamous cell carcinoma who underwent staging work-up which demonstrated additional asymptomatic solitary lung and pancreatic masses, raising the diagnostic dilemma of metastatic disease, and what type of primary malignancy, versus synchronous primary malignancies. Metastasis can occur by three main principal mechanisms: direct extension, lymphatic spread, or hematogenous spread (6). In esophageal squamous cell cancer, the most common sites of metastasis are lung, lymph nodes, liver, and bone (7). In contrast, according to autopsy registries, metastasis to the esophagus from another primary malignancy is a rare phenomenon with incidences ranging from 0.3% to 6.1% (8, 9). In these rare cases, direct extension is the most common etiology (10). Regarding pancreatic cancer, the majority of pancreatic malignancies, representing approximately 90%, are primary adenocarcinomas, exocrine in origin (11). The remaining 10% of pancreatic malignancies include squamous cell carcinoma, small cell carcinoma, acinar cell carcinoma, and metastatic lesions (12).

Given our patient’s presentation with the classical symptoms for esophageal cancer with progressive dysphagia and weight loss, a small left lower lobe pulmonary nodule with no evidence of direct extension, and a large pancreatic tail mass with low likelihood to be squamous in origin given less than 1% of pancreatic cancers are primary squamous cell (13), our initial differential diagnosis was metastatic esophageal squamous cell carcinoma with synchronous primary pancreatic cancer. Biopsies of both the lung nodule and the pancreatic mass were obtained and demonstrated cells with similar malignant-appearing epithelial cells with a squamous morphology infiltrating into surrounding tissue. These biopsies (lung, pancreatic) were subsequently compared to the original biopsy of the esophageal mass and the malignant cells were histologically similar (**Figure 2**). Consequently, we favored a diagnosis of primary esophageal squamous cell carcinoma, metastatic to lung and pancreas. To attempt to confirm this diagnosis, umor origin test was performed where RNA expression data is analyzed to determine the histological subtype of the malignancy (4). Novel molecular diagnostic classifiers such as the Tempus Tumor Origin test have been retrospectively used to assess the accuracy of cancers of uncertain primary, demonstrating that these assays could affect treatment in the majority of patients (14). However, further prospective studies are needed to assess if these molecular classifiers improve outcomes in the setting of uncertainty about the primary. Treatment was initiated with chemotherapy and immunotherapy but was discontinued after 3 cycles of treatment given rapidly progressive disease. The patient transitioned to hospice.

Pancreatic metastases are uncommon, accounting for approximately 2-5% of pancreatic tumors (2). Though any tumor type has the capacity to metastasize to the pancreas, the majority are renal cell carcinoma in origin, accounting for up to 70% of cases, followed by melanoma, colorectal cancer, sarcoma, breast cancer, and lung cancer, all representing single-digit percentage incidences (15). Pancreatic metastases can develop synchronously or develop meta-synchronously. Most pancreatic metastases are asymptomatic and found incidentally on imaging for staging purposes and can be difficult to differentiate between a primary pancreatic tumor and a pancreatic metastasis, or at autopsy (16). If symptoms are present, likely if the metastasis is to the head of the pancreas, the most common symptoms are epigastric and/or back pain, anorexia, weight loss, nausea, vomiting, gastrointestinal hemorrhage, and jaundice (17). Biopsy of the pancreatic mass is necessary to distinguish between pancreatic metastasis and primary pancreatic cancer, though metastases may cytologically mimic primary pancreatic cancers (18).

In terms of treatment of pancreatic metastases, there is evolving literature, where the primary malignancy is renal cell carcinoma, and in well-selected cases, surgical resection can provide survival benefits (19). In our case, esophageal squamous cell carcinoma was the primary. To the best of our knowledge, fewer than 10 cases of pancreatic metastasis of esophageal squamous cell carcinoma have been reported (3). Most patients with esophageal squamous cell carcinoma metastatic to the pancreas are not surgical candidates given the widespread nature of their disease, and prognosis is universally poor, matching that of the metastatic primary.

In summary, esophageal squamous cell carcinoma metastatic to the pancreas is extremely rare. In terms of clinical implications for future presentations of esophageal squamous cell carcinoma metastatic to the pancreas, diagnosis requires biopsy with histopathologic confirmation, treatment options are limited by the extent of disease and rarity of the presentation with systemic therapy being the mainstay, and indicate poor prognosis as consistent with advanced, disseminated disease, potentially influencing the choice or aggressiveness of therapy

### Learning points

- Esophageal squamous cell carcinoma typically metastasizes to the lungs, liver, and bone.
- Pancreatic metastases are rare and poorly understood manifestations of esophageal squamous cell carcinoma. Histopathologic confirmation is critical.
- Limited treatment options with systemic therapy the mainstay.
- Prognosis is universally poor.

## Data Availability

The original contributions presented in the study are included in the article/supplementary material. Further inquiries can be directed to the corresponding author.
